# Non-invasive assessment of atherosclerotic coronary lesion length using multidetector computed tomography angiography: comparison to quantitative coronary angiography

**DOI:** 10.1007/s10554-012-0015-7

**Published:** 2012-01-24

**Authors:** J. E. van Velzen, M. A. de Graaf, A. Ciarka, F. R. de Graaf, M. J. Schalij, L. J. Kroft, A. de Roos, J. W. Jukema, J. H. C. Reiber, J. D. Schuijf, J. J. Bax, E. E. van der Wall

**Affiliations:** 1 Department of Cardiology, Leiden University Medical Center, Postal Zone: C5-P, P. O. Box 9600, 2333 ZA Leiden, The Netherlands; 2 The Interuniversity Cardiology Institute of The Netherlands, Utrecht, The Netherlands; 3 Department of Cardiovascular Diseases, University Hospital Gasthuisberg, Catholic University Leuven, Leuven, Belgium; 4 Department of Radiology, Leiden University Medical Center, Leiden, The Netherlands

**Keywords:** Coronary artery disease, Multidetector computed tomography, Quantitative coronary angiography

## Abstract

Multidetector computed tomography angiography (CTA) provides information on plaque extent and stenosis in the coronary wall. More accurate lesion assessment may be feasible with CTA as compared to invasive coronary angiography (ICA). Accordingly, lesion length assessment was compared between ICA and CTA in patients referred for CTA who underwent subsequent percutaneous coronary intervention (PCI). 89 patients clinically referred for CTA were subsequently referred for ICA and PCI. On CTA, lesion length was measured from the proximal to the distal shoulder of the plaque. Quantitative coronary angiography (QCA) was performed to analyze lesion length. Stent length was recorded for each lesion. In total, 119 lesions were retrospectively identified. Mean lesion length on CTA was 21.4 ± 8.4 mm and on QCA 12.6 ± 6.1 mm. Mean stent length deployed was 17.4 ± 5.3 mm. Lesion length on CTA was significantly longer than on QCA (difference 8.8 ± 6.7 mm, *P* < 0.001). Moreover, lesion length visualized on CTA was also significantly longer than mean stent length (CTA lesion length-stent length was 4.2 ± 8.7 mm, *P* < 0.001). Lesion length assessed by CTA is longer than that assessed by ICA. Possibly, CTA provides more accurate lesion length assessment than ICA and may facilitate improved guidance of percutaneous treatment of coronary lesions.

## Introduction

Invasive coronary angiography (ICA) has been traditionally used for evaluation of the presence and severity of coronary artery disease (CAD). Accordingly, the technique has been extensively utilized to guide further treatment strategies, such as percutaneous coronary intervention (PCI) with stent placement. In particular, the choice for stent length and diameter is frequently decided on the basis of the 2-dimensional ICA images. However, although ICA has an excellent ability to visualize the lumen and severity of luminal narrowing, the presence of atherosclerotic plaque in the arterial wall cannot be accurately visualized [1]. The chosen stent length may not always match the true atherosclerotic plaque length and could potentially lead to insufficient stent coverage of the plaque and possible development of post-stent complications such as arterial dissection, in-stent restenosis and stent thrombosis [2–4].

Several studies comparing ICA to intravascular ultrasound (IVUS) have shown that ICA indeed underestimates plaque extent [1, 5, 6]. Multidetector computed tomography angiography (CTA) is increasingly used to non-invasively evaluate the presence of CAD [7, 8], and a growing number of patients referred for ICA will have previously undergone non-invasive evaluation by CTA. A particular strength of this modality is that it is able to not only visualize luminal narrowing but also the extent of atherosclerotic plaque in the arterial wall [9, 10]. Accordingly, in patients with previous CTA, who subsequently underwent ICA and PCI, lesion length on CTA was compared to length on ICA.

## Methods

### Patients

A total of 89 patients were retrospectively analyzed, who were clinically referred for CTA and had subsequent ICA and PCI with stent implantation. For this retrospective evaluation, consecutive patients were selected as part of an ongoing registry addressing the relative merits of CTA in relation to other imaging techniques [11]. All clinical data were retrieved from the departmental Cardiology Information System (EPD-Vision^®^, Leiden University Medical Center). In each patient, the presence of CAD risk factors such as diabetes, systemic hypertension, hypercholesterolemia, positive family history, smoking and obesity, were noted.

### Multidetector computed tomography coronary angiography

#### Data acquisition

Contra-indications for CTA were (1) (supra) ventricular arrhythmias, (2) renal insufficiency (glomerular filtration rate < 30 ml/min), (3) known allergy to iodine contrast material, (4) severe claustrophobia, (5) pregnancy.

Patients received beta-blocking medication (50–100 mg metoprolol orally, or 5–10 mg intravenously) 1 h before CTA examination if the heart rate was above 65 beats per minute, unless contra-indicated. Forty-seven patients were scanned on a 64-detector row helical scanner (Aquilion 64, Toshiba Medical Systems, Otawara, Japan). Scan parameters were: 400 ms gantry rotation time, 100–135 kV tube voltage and a tube current of 250–350 mA, depending on body shape. Thirty-six patients were scanned on a 320-detector row volumetric scanner (Aquilion ONE, Toshiba Medical Systems, Otawara, Japan). The heart was imaged in a single heartbeat, using prospective triggering with exposure interval depending on the heart rate. Scan parameters were: 350 ms gantry rotation time, 100–135 kV tube voltage and a tube current of 400–580 mA, depending on body mass index. In total, 60–90 ml contrast material (Iomeron 400, Bracco, Milan, Italy) was administered with a rate of 5–6 ml/s followed by a saline flush. Subsequently, data sets were reconstructed in the best available phase and transferred to a remote workstation.

#### CTA lesion length assessment

**Fig. 1:**
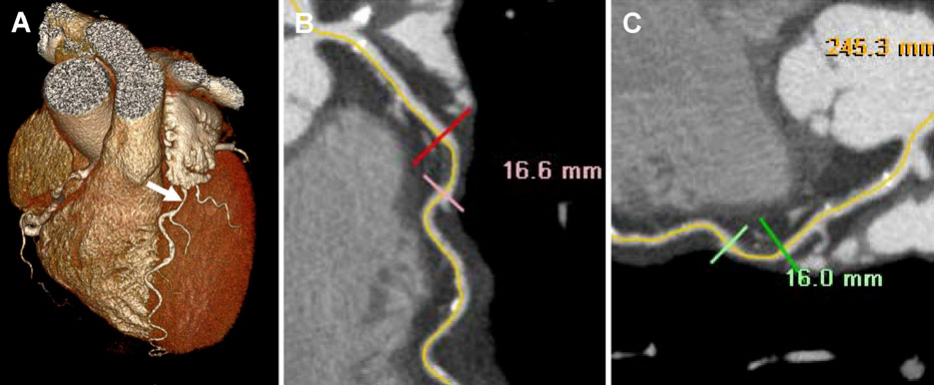
Example of atherosclerotic lesion length measurement in two different views on multidetector computed tomography angiography images with the use of a dedicated software tool. In (**a**), a 3 dimensional volume rendered reconstruction of the heart with the left anterior descending coronary artery (LAD) is shown (*arrow*). In (**b**) lesion length measurement is performed of a non-calcified lesion in the mid LAD. In this view, lesion length measured was 16.6 mm. In (**c**), lesion length measurement is performed of the same lesion, however in a different angle. In this view, lesion length measured was 16.0 mm

Post-processing of the CTA scans was performed on a dedicated workstation (Vitrea FX 2.0.2, Vital images Minnetonka, MN, USA). Coronary anatomy was assessed in a standardized manner by dividing the coronary artery tree into 17 segments according to the modified American Heart Association classification. CTA lesion length was evaluated in consensus by 2 experienced readers who were blinded to quantitative coronary angiography (QCA) lesion length findings. Firstly, the location of lesions was identified on ICA. To match lesions identified on ICA with lesions on CTA, landmarks such as coronary ostia, side-branches and calcium deposits were used. A plaque on CTA was defined as a structure ≥1 mm^2^ in the coronary artery lumen [12]. Secondly, on CTA, lesion length was determined on curved multiplanar reconstructions (MPR’s) in two different angles for every lesion in which PCI was performed. Lesion length (mm) was measured on CTA from the proximal to distal shoulder of the plaque with a dedicated Vitrea software display tool (Vitrea FX 2.0.2, Minnetonka, MN, USA). A tandem lesion within 4 mm of the edge of lesion was considered as part of the lesion. The average of these two measurements was taken as the final CTA lesion length. An example of lesion length measurement on CTA is shown in Fig. 1. Radiation dose was quantified with a dose-length product conversion factor of 0.014 mSv/(mGy cm) [13]. For the 320-row CTA, patients with a low heart rate (<60 bpm) were scanned full dose at 70–80% of R-R interval and estimated mean radiation dose was 3.2 ± 1.1 mSv. Patients with a higher heart rate (60–65 bpm) were scanned full dose at 65–85% of R–R interval and estimated mean radiation dose was 7.1 ± 1.7 mSv. For the 64-row CTA, the estimated mean radiation dose was 18.1 ± 5.9 mSv, all performed with retrospective gating.

### Quantitative coronary angiography

Quantitative coronary angiography analysis was performed by observer unaware of CTA findings with the use of QCA-CMS version 6.0 (Medis, Leiden, The Netherlands). Prior to measuring lesion length, images were calibrated with use of the contrast filled catheter. Subsequently, per lesion, the two best orthogonal projections were chosen on which measurements were performed to minimize foreshortening. Consequently, lesion length (mm) was measured from the proximal shoulder to the distal shoulder of the lesion. The longest length measured on QCA was used for further analysis. In addition, highest percent diameter stenosis as measured by QCA was reported for each lesion. The choice and size of stent used were left to the discretion of operator. Per lesion, stent diameter and length were reported. If more than one stent was planned, the total stent length of all combined stents deployed was used.

### Statistical analysis

Continuous variables were expressed as mean and standard deviation, and categorical data were expressed in numbers and percentages. Paired variables were analyzed with Wilcoxon signed rank tests. Statistical analysis was performed using SPSS software (version 16.0, SPSS Inc., Chicago, IL, USA). A *P* value < 0.05 was considered statistically significant.

## Results

Of the 89 patients, 4 were excluded in which the target lesion length was not quantifiable on CTA. These lesions were either a total chronic occlusion (n = 2) or in-stent restenosis (n = 2). Furthermore 2 patients were excluded due to impaired CTA image quality. Consequently, 83 patients (59 males (71%), mean age 62 ± 10 years) and 119 lesions were included in the analysis. Baseline patient characteristics are described in Table 1. The average time interval between CTA and PCI was 63 ± 110 days. As determined by QCA, the mean percent stenosis of the lesions was 71 ± 11%. Overall, lesions were most often located in the left anterior descending coronary artery (56 lesions, 47%) followed by the left circumflex coronary artery (35 lesions, 29%) and the right coronary artery (28 lesions, 24%). Concerning plaque composition, 27 lesions (23%) were non-calcified, 72 lesions (60%) were mixed and 20 lesions (17%) were calcified.

**Table 1:** Patient characteristics (n = 83)

	n (%)
Age (mean ± SD)	62 ± 10
Gender (male/female)	59/24
Obesity (BMI ≥ 30 kg/m^2^)	12 (14%)
Diabetes	18 (22%)
Hypertension	44 (53%)
Hypercholesterolemia	28 (34%)
Family history	37 (45%)
Smoking	33 (40%)
Previous stent (%)	18 (22%)
Complications during PCI	
Edge dissection	6 (7%)
Stent thrombosis^a^	1 (1%)

*BMI* body mass index, *PCI* percutaneous coronary intervention

^a^Defined as definite, probable and possible stent thrombosis within 1 month

### Lesion length

**Fig. 2:**
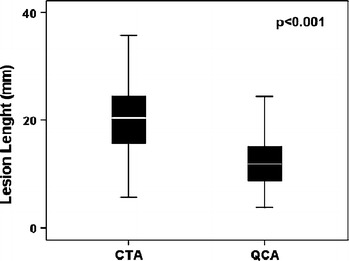
Box plot showing the difference between lesion length assessment on multidetector computed tomography angiography (CTA) and quantitative coronary angiography (QCA). Lesion length assessment is less on QCA as compared to CTA (*P* < 0.001)

**Fig. 3:**
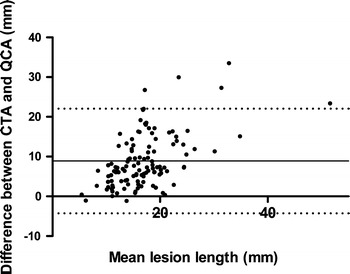
Bland–Altman plot of lesion length (mm) shows the difference between each pair plotted against the average value of the same pair (*solid line* mean value of difference, *dotted line* mean value of differences ± 2 SDs)

The mean lesion length measured on CTA was 21.4 ± 8.4 mm, as compared to a mean lesion length on QCA of 12.5 ± 6.1 mm resulting in a mean difference (CTA lesion length − QCA lesion length) of 8.8 ± 6.7 mm. Only 2 lesions were longer on QCA than on CTA, however the difference between these 2 lesions was only minor (13.2 mm on CTA vs. 14.3 mm on QCA and 5.7 mm on CTA vs. 6.7 mm on QCA). Overall, mean CTA lesion length was significantly longer than mean QCA lesion length (*P* < 0.001), as demonstrated in Fig. 2. At Bland–Altman analysis, mean differences (±SD) of 8.8 ± 6.7 mm were observed between CTA and ICA, with 95% limits of agreement ranging from −4.3 to 22.0 (Fig. 3). The mean diameter of stents deployed was 3.1 ± 0.3 mm, ranging from 3.0 to 4.0 mm. The length of stents deployed ranged from 8.0 to 33.0 mm (mean 17.5 ± 5.3 mm). Accordingly, lesion length on CTA was also significantly longer than mean stent length (CTA lesion length − stent length was 4.2 ± 8.7 mm, *P* < 0.001). Furthermore, mean stent length exceeded lesion length on QCA; stent length − QCA lesion length was 4.8 ± 6.2 mm (*P* < 0.001).

## Discussion

Comparison between ICA and non-invasive CTA demonstrated that lesion length measured by CTA was substantially longer than lesion length measured by ICA. In addition, lesion length on CTA was compared to the stent length selected for PCI. Interestingly, lesion length measured by CTA significantly exceeded the mean length over which the stent was deployed.

There are only very limited data regarding lesion length measurement with CTA. Soon et al. [14] compared atherosclerotic lesion length between ICA and CTA (16-slice) in 30 patients and 44 lesions and observed that lesion length was significantly longer on CTA than on ICA with a median difference of 9.8 mm (95% confidence interval of 7.3–13.3). Moreover, the finding that ICA significantly underestimates atherosclerotic lesion length has also been demonstrated by Yamagishi et al, who compared atherosclerotic lesion length on ICA to grayscale IVUS, the current gold standard for the evaluation of atherosclerosis [5]. The authors demonstrated that lesion length on ICA (mean lesion length of 12.4 ± 6.1 mm) was significantly shorter than lesion length measured by IVUS (mean lesion length of 16.3 ± 8.9 mm).

Although angiographically detected coronary atherosclerosis has been linked to outcome in several clinical trials [15–18], it has been suggested that ICA considerably underestimates the overall extent of CAD [19]. Indeed, when compared to IVUS, the presence of angiographic disease did not reflect true atherosclerotic plaque burden [1]. Mintz et al. [1] compared the detection of atherosclerosis on ICA to IVUS and found that only 7% of segments described as normal on ICA were truly without any atherosclerotic plaque on IVUS. Conversely, non-invasive CTA has been shown to provide accurate evaluation of coronary plaque burden [9, 20–22], with a good correlation for detecting and characterizing atherosclerotic plaque in comparison to histology [23–25]. Moreover, Leber et al. [12] demonstrated a sensitivity and specificity of 16-slice CTA for the detection of coronary lesions as determined on IVUS of 85 and 92%, respectively. However, small plaques located in distal segments were more difficult to evaluate, particularly in CTA scans with reduced image quality [12].

The large difference between ICA and CTA lesion length assessment can be explained by the excellent ability of CTA to visualize plaque and vessel remodelling in the arterial wall, in contrast to ICA. Indeed, ICA only shows the contrast filled lumen and is unable to visualize the arterial wall (with the exception of large calcifications), and reference segments may not be optimally evaluated by ICA [26]. Indeed, with traditional invasive coronary angiography, lesions with outward (positive) remodeling are frequently underestimated or missed. Moreover, CTA is not hampered by limitations of angiographic projection such as foreshortening or difficulties in case of tortuous vessels. However, it is of importance to identify the presence of atherosclerosis even at sites without significant luminal narrowing, and some acute coronary syndromes may be triggered by sudden disruption of atherosclerotic plaques that caused neither significant luminal narrowing nor chest pain complaints before the event [27]. It has been suggested that the most rupture prone part of the plaque is not at the maximum point of luminal narrowing but actually located in the shoulders of the plaque [28]. Accordingly, it is important to assess the entire plaque length.

It has also been demonstrated that insufficient coverage of the target lesion increases the risk for in-stent restenosis and stent thrombosis. Fujii et al. retrospectively evaluated lesion characteristics using IVUS leading to stent thrombosis after PCI [2]. The authors observed that stent thrombosis occurred in stents with significantly more residual plaque upstream and downstream from the stent as compared to lesions without stent thrombosis. Okabe et al. [3] explored which IVUS related findings predicted the risk for the development of stent thrombosis. The authors observed that residual disease at the edge of the stent was associated with subsequent stent thrombosis. Of note, a higher residual plaque burden proximal from the stent has also been associated with increased rates of in-stent restenosis [4]. Accordingly, optimal assessment of the lesion length may improve success and outcome of interventional procedures [29].

### Limitations

Several limitations need to be addressed. First, there was a time-interval between CTA and PCI during which atherosclerosis may have progressed. Second, only lesions subsequently treated by PCI were included in the study. Therefore, the current observations are only based on lesions with a high grade stenosis. Third, in the current study QCA was not part of the clinical routine of the laboratory and the difference between the CTA lesion length and the stent length chosen reflects the discrepancy between stent length driven by visual assessment. Furthermore, the attending interventional cardiologist did not incorporate lesion topography on CTA before performing PCI. However, future studies should be performed to evaluate the value and improved outcome of CTA lesion assessment prior to PCI.

## Conclusion

Lesion length assessed by CTA is longer than lesion length assessed by ICA, which may facilitate improved guidance of percutaneous treatment of coronary lesions.
